# Understanding Athlete Emotions: A Psychometric Approach to the AEQ-S in Sports

**DOI:** 10.3390/brainsci16010046

**Published:** 2025-12-29

**Authors:** María-Jesús Lirola, Rubén Trigueros, José Manuel Aguilar Parra, Clemente Franco

**Affiliations:** Department of Psychology, Health Research Centre, University of Almería, 04120 Almería, Spain; mariajesus.lirola@ual.es (M.-J.L.); jmaguilar@ual.es (J.M.A.P.); cfranco@ual.es (C.F.)

**Keywords:** achievement emotions questionnaire, athlete, emotion, resilience, sport

## Abstract

Introduction: This study focuses on the adaptation and validation of the Achievement Emotions Questionnaire-Short (AEQ-S) to the Spanish sports context. Emotions play a crucial role in athletes’ decision making, making it essential to have reliable assessment tools tailored to this field. Method: The AEQ-S was administered to 998 professional athletes (mean age: 26.83 years). The adaptation followed the Hambleton method and involved the support of sports psychologists. Exploratory (EFA) and confirmatory factor analyses (CFAs) were conducted, along with tests for reliability and criterion validity. Results: The analyses confirmed that the factor structure of the AEQ-S in sports aligns with the original version, identifying eight key emotions: enjoyment, hope, pride, anxiety, anger, shame, hopelessness, and boredom. Furthermore, positive emotions were positively associated with resilience, while negative emotions showed an inverse relationship. Conclusions: The adapted AEQ-S proved to be a valid and reliable tool for assessing emotions in athletes. Its applications extend to both research and professional practice in the sports domain.

## 1. Introduction

In sport settings, emotions are not only pervasive but are also fundamentally shaped by the achievement-oriented nature of practice and competition. Achievement emotions in sport are embedded in contexts characterised by immediate performance feedback, public evaluation, physical demands, and competitive or self-referenced standards, which distinguish them conceptually from those experienced in academic environments [1]. These emotions arise from athletes’ appraisals of success, failure, control, and goal attainment, and they may differ in both intensity and functional significance from achievement emotions in educational settings. For example, positive emotions such as enjoyment, pride, or hope may enhance motivation, persistence, and performance during training or competition, whereas negative emotions such as anxiety, anger, shame, or hopelessness can undermine concentration, impair decision making, and increase the risk of disengagement or dropout [2,3]. Importantly, the antecedents and consequences of these emotions in sport are closely linked to sport-specific factors, including competitive pressure, coach–athlete interactions, and the embodied nature of performance. Despite these contextual particularities, empirical research has often relied on instruments originally developed for academic settings, such as the Achievement Emotions Questionnaire (AEQ), underscoring the need to examine the applicability and validity of sport-adapted versions like the AEQ-S within athletic populations.

This conceptualization of sport as an achievement context is grounded in the shared structural characteristics it has with the academic environment. According to the Control-Value Theory [4], both domains are defined by activities where outcomes are judged against standards of excellence. In sports, as in academia, athletes are subject to evaluative situations—such as training sessions (analogous to learning settings) and competitions (analogous to exams)—that trigger specific emotions based on the appraisals of control and value. While the objects of these emotions differ (technical mastery vs. academic grades), the underlying psychological mechanism is identical. Consequently, the experience of all these emotions in the sports context is related to other variables such as motivation, learning, performance, and the health of the participants [5]. Furthermore, it significantly contributes to the development of athletes’ identity [6]. In this regard, the emotions experienced on the playing field influence how athletes perceive themselves, impacting their sports identity and their role within the team [7]. Given the importance and significance of the emotional connection with these aspects considered fundamental for well-being, sports performance, and personal development, there is an interest in having adequate and rigorous psychometric tools. These tools should be developed under a theoretical framework that supports them, capable of assessing these emotions in physical-sports contexts. To date, there are no specific assessment instruments for the sports context; however, a self-report instrument can be found that measures various achievement-related emotions in an educational context. This instrument assesses the emotions commonly experienced by students in academic settings (Achievement Emotions Questionnaire, AEQ; [8]).

The AEQ was created and developed under the knowledge framework established in the Control-Value Theory of achievement emotions [4], using this as the basis for defining the emotions, constructing the scales (currently using an abbreviated version of the AEQ called AEQ-S; [9]) and their validation. The theory of control-value posits that emotions of control and value experienced in various contexts, where an individual is required to intervene or participate, facilitate the induction of achievement-related emotions [8]. In this regard, achievement contexts are defined as situations or life areas in which a person’s involvement leads to successful outcomes, encompassing academic environments and achievement situations in other areas of life, such as sports or professional activities [4]. This theory draws from different approaches, including theories that consider cognition and emotion as mediators of learning and achievement, as well as achievement expectations [10]. Additionally, it integrates elements from transactional approaches that address the role of stress, appraisal, and coping processes [11], as well as attributional theories of motivation and emotion [12]. It also incorporates approaches explaining models of the effects of emotions on performance, as proposed by Fredrickson [13], Pekrun [14], and Zeidner [15].

The Control-Value Theory (CVT) of achievement emotions provides a robust framework to explain how emotions experienced during an activity are preceded by feelings of control over the situation and subjective appraisals of achievement [14]. Within this framework, emotions are classified through a taxonomy based on three dimensions: valence (positive or negative), activation (activating or deactivating), and object focus (directed toward the process or the outcome, such as success or failure) [8]. While CVT originated in educational settings, its integration into the sports domain is highly consistent with established models such as the Biopsychosocial Model of Stress (BPSM) [16] and the Individual Zones of Optimal Functioning (IZOF) [17]. Specifically, the ‘control’ appraisal in CVT mirrors the evaluation of resources versus demands in the BPSM, suggesting that both frameworks converge on how athletes interpret achievement settings. Furthermore, while CVT explains the cognitive origins of these emotions, the IZOF model complements this by emphasising how these emotional experiences must fall within specific individualised ranges to optimise athletic performance [18]. Thus, the synergy between these theories explains the relationship between an activity’s control-value, the resulting emotional experience, and the subsequent performance outcomes.

Previous studies have included analyses of the instrument in preliminary or partial factor selection versions of the scale without applying it in its entirety [8,19,20,21]. However, it was Pekrum et al. [14] who conducted the first comprehensive investigation of the AEQ, encompassing all scales of the instrument in a single analysis. Since then, research conducted with the AEQ has demonstrated its reliability and validity, both internally and externally, supporting its use in academic and school contexts. Additionally, adaptations of the instrument have been developed for various subjects such as mathematics or physical education [22,23], different educational stages, whether in primary or university education [21,24], and in various languages, including Italian, American, or German [24,25], among others. The hypotheses put forward in the Control-Value Theory of achievement emotions are thus refuted [14].

The AEQ will assess achievement emotions from a multicomponent perspective. The current shortened version, the AEQ-S [9], has managed to reduce its length and facilitate its applicability, consisting of 24 items, spread over three different academic contexts such as learning, classes, and exams. This tool takes into account the characteristics of the context in its influence on the perception of control and value, and the emotions of achievement experienced. Although the Achievement Emotions Questionnaire (AEQ) was originally developed for academic contexts, its underlying theoretical framework—the value-control model of achievement emotions—is applicable to sport. Both academic and sporting environments involve goal-oriented activities, performance evaluation, and subjective assessments of success and failure, which elicit emotions that influence motivation, commitment, and well-being. However, sport introduces context-specific factors, such as immediate feedback, physical demands, social evaluation by coaches and teammates, and competitive pressures, which shape the antecedents and consequences of achievement emotions [26,27]. Therefore, the AEQ-S represents a theoretically grounded adaptation that captures the same fundamental constructs while reflecting the distinctive dynamics of sports environments. Consequently, the AEQ-S provides a validated instrument for measuring achievement emotions in athletes, enabling researchers and professionals to systematically assess emotional experiences during training and competition. Thus, the adaptation of this instrument to the sport context is hypothesised to allow the assessment of achievement emotions in learning contexts, training sessions, and competition.

The main objective of this study would be to adapt the short version of the AEQ-S designed and validated by Bieleke et al. [9] in an academic context with university students to the sport context. The hypotheses of the study are as follows:

**H1** **(Factorial validity).** *It is hypothesised that the AEQ-S will demonstrate an adequate factorial structure in the sport context, as evidenced by acceptable fit indices in a confirmatory factor analysis (χ^2^/df < 3.0, CFI and TLI ≥ 0.95, and RMSEA and SRMR ≤ 0.06).*

**H2** **(Internal consistency reliability).** *It is hypothesised that all AEQ-S subscales will show satisfactory internal consistency, with Cronbach’s alpha and composite reliability coefficients equal to or greater than 0.70.*

**H3** **(Convergent validity).** *It is hypothesised that the AEQ-S factors will exhibit adequate convergent validity, as indicated by standardised factor loadings ≥ 0.50 and average variance extracted (AVE) values ≥ 0.50.*

**H4** **(Discriminant validity).** *It is hypothesised that the AEQ-S will demonstrate discriminant validity, such that the square root of the AVE for each factor exceeds the inter-factor correlations.*

## 2. Materials and Methods

### 2.1. Participants

The programme G*Power 3.1 [28] was used to calculate the sample size. The parameters used for the calculation were α = 0.05 and statistical power (1 − β) = 0.80. Using these data, the minimum sample size was 251 athletes. Therefore, the sample of 998 athletes for this study was considered sufficient.

For the selection of the 998 athletes, non-random sampling was used, in accordance with the criteria of Levy & Lemeshow (see [29]). However, in order to divide the sample according to the different analyses, which are detailed below, a randomization analysis was used through SPSS. 33.77% of the participants practice soccer, 25.15% basketball, 19.94% volleyball, 11.42% handball, and 9.72% athletics.

A total of 468 professional athletes were used for the CFA. The age of the athletes ranged from 17 to 35 years (M = 25.61, SD = 5.88). The sample population is divided into 217 males and 251 females.

A total of 331 professional athletes were used to conduct the EFA. The age of the athletes ranged from 18 to 34 years (M = 27.54, SD = 5.30). The sample population is divided into 181 males and 150 females. Their training experience ranged from 5 to 19 years, with an average training frequency of four sessions per week.

For the analysis of temporal stability, through the test–retest, the participants were 199 athletes (103 males and 96 females), from 19 to 33 years, who completed the instrument at two different times, separated by two weeks. Their training experience ranged from 5 to 18 years, with an average training frequency of four sessions per week.

### 2.2. Measurements

Sport Emotions. To analyse students’ emotions in a sports context, the factor structure of the AEQ-S of Bieleke et al. [29] was adapted and tested. The AEQ-S is divided into three subscales: Emotions related to learning, to classes, and to exams. However, these factors have been renamed to refer to the sport context (sport learning (Appendix A), training sessions (Appendix B), and competition (Appendix C)). Each of the emotion subscales is made up of eight emotions (enjoyment, anger, pride, anxiety, hope, hopelessness, embarrassment, and boredom/relief). Athletes responded to each of the scales using a Likert-type format where 1 is strongly disagree and 5 is strongly agree.

Resilience in Sport Context. To analyse this factor, the Resilience in Sport Context Scale (ERCD, [30]) was used. The scale is divided into two sub-scales and 25 grouped items: 17 of which correspond to personal competence and 8 to acceptance of self and life. Athletes responded to each of the scales using a Likert type format where 1 = Disagree and 7 = Strongly Agree.

### 2.3. Procedure

To initiate the research, each AEQ-S item had to be converted into Spanish. This was accomplished using Hambleton’s [31] direct and back-translation method. Initially, two translators with expertise in sports psychology rendered the items into Spanish. Subsequently, a different team of three translators retranslated these Spanish items back into English. The resulting English version was determined to be comparable to the original questionnaire. Following translation, three sports psychologists with over a decade of experience conducted an expert review to ensure content validity. The experts evaluated each item based on three fundamental criteria: relevance (the degree to which the item measures the intended emotion of achievement), clarity (comprehensibility of the language for athletes of different ages), and representativeness (suitability of the item to the training or competition context). The items included in the AEQ-S achieved unanimous consensus on their cultural and semantic relevance in the Spanish sporting context. To mitigate the risk of cultural bias inherent in linguistic adaptation, the translation process was not purely literal, but focused on conceptual equivalence.

Once the final questionnaire was obtained, 12 provincial federations (from Almeria, Murcia, Granada, and Valencia) and 47 sports clubs (from Almeria, Murcia, Granada, and Valencia) were contacted to request their collaboration in order to establish contact with the athletes, and a positive response was obtained from 100% of the federations and 85.1% of the clubs. Once they had been contacted, the informed consent form was given to the athletes so that they could fill it in. Once the document had been handed in, the questionnaire was administered to the athletes at the start of the training session, in paper format and individually. Before completing the questionnaires, it was emphasised that participation was voluntary and that their answers would be confidential.

This study has been approved by the bioethics committee of the University of Almería in order to start the present study (Ref. UALBIO2025/035). In addition, the protocols established by the American Psychology Associations and the Helsinki Declaration have been respected at all times.

### 2.4. Data Analysis

The AMOS v21 and SPSS v23 statistical packages were used to analyse the factor structure and reliability of the AEQ-S in the sport context. In this way, three EFAs were applied to determine the behaviour of the items in each proposed factor. For the EFA, KMO test indices located between 0.5 and 1 can consider that the factor structure is adequate [32]. Subsequently, three CFAs were carried out to test the factor structures of each of the scales. Finally, the reliability of the questionnaire was analysed using Cronbach’s alpha, the omega coefficient, an analysis of temporal stability through a retest and linear regression.

The method used for the CFA was the maximum likelihood estimation method, as this method takes into account the non-normal distribution of the data and is also recommended when using Likert-type questionnaires [33]. In addition to this method, the bootstrapping standard errors procedure of 6000 replications was used. Despite the non-normality, the estimators were not affected and were therefore considered robust [34]. The criteria set by Hair et al. [35] were applied to determine whether to accept or reject the factor structure of the scales, considering the following estimators: RMSEA and the SRMR, showing a good fit as long as the score is equal to or below 0.06; the incremental indices (IFI, CFI, and TLI) show a good fit as long as the score is above 0.95; and the χ^2^/df, showing a good fit as long as the score is between 2 and 3.

## 3. Results

### 3.1. Exploratory Factorial Analysis

The analyses used in the EFA reflected that the scores obtained in the Kaiser–Meyer–Olkin test (KMO = 0.96) and Bartlett’s statistics (χ^2^ (496) = 4744, *p* < 0.001) had acceptable fit indices for the emotions linked to learning. The results obtained in the exploratory factor analysis are shown in Table 1.

The analyses employed in the EFA reflected that the scores obtained in the Kaiser–Meyer–Olkin test (KMO = 0.96) and Bartlett’s statistics (χ^2^ (496) = 4632, *p* < 0.001) had acceptable fit indices for emotions during training. Table 2 shows the results obtained in the exploratory factor analysis.

The analyses employed in the EFA reflected that the scores obtained in the Kaiser–Meyer–Olkin test (KMO = 0.95) and Bartlett’s statistics (χ^2^ (496) = 4587, *p* < 0.001) had acceptable fit indices for emotions during competition. Table 3 shows the results obtained in the exploratory factor analysis.

### 3.2. Confirmatory Factor Analysis

**Figure 1:**
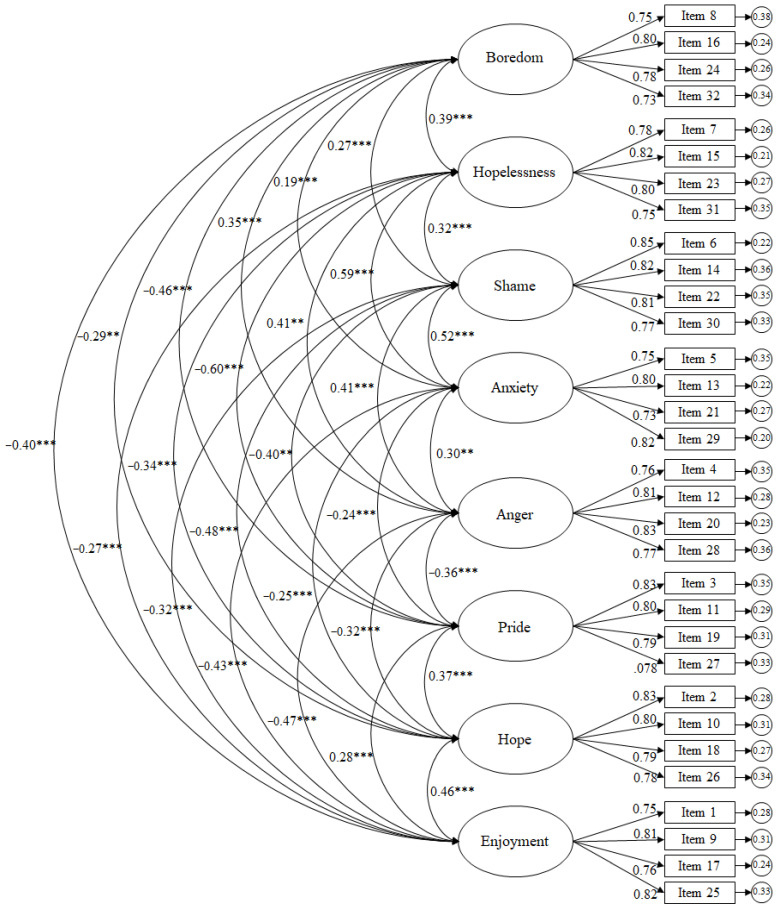
CFA of the AEQ-S focused on the learning context. The ellipses represent the factors and the rectangles represent the different items. The residual variances are shown in the small circles. Note: *** *p* < 0.001; ** *p* < 0.01.

Results reflected in the CFA for the training-related emotions scale revealed the following scores (Figure 1): χ^2^ (436, N = 468) = 934.69, *p* = 0.001; χ^2^/df = 2.14; CFI = 0.96; TLI = 0.96; IFI = 0.96; RMSEA = 0.052 (95% CI = 0.047–0.056); SRMR = 0.040. To evaluate local fit, standardised residuals were examined, revealing that all absolute values were below 2.58, indicating no major item-level misfit. The factor correlation matrix showed moderate correlations between factors, supporting discriminant validity while remaining consistent with the theoretical model. Standardised regression weights were statistically significant (*p* < 0.001), ranging from 0.75 to 0.85.

**Figure 2:**
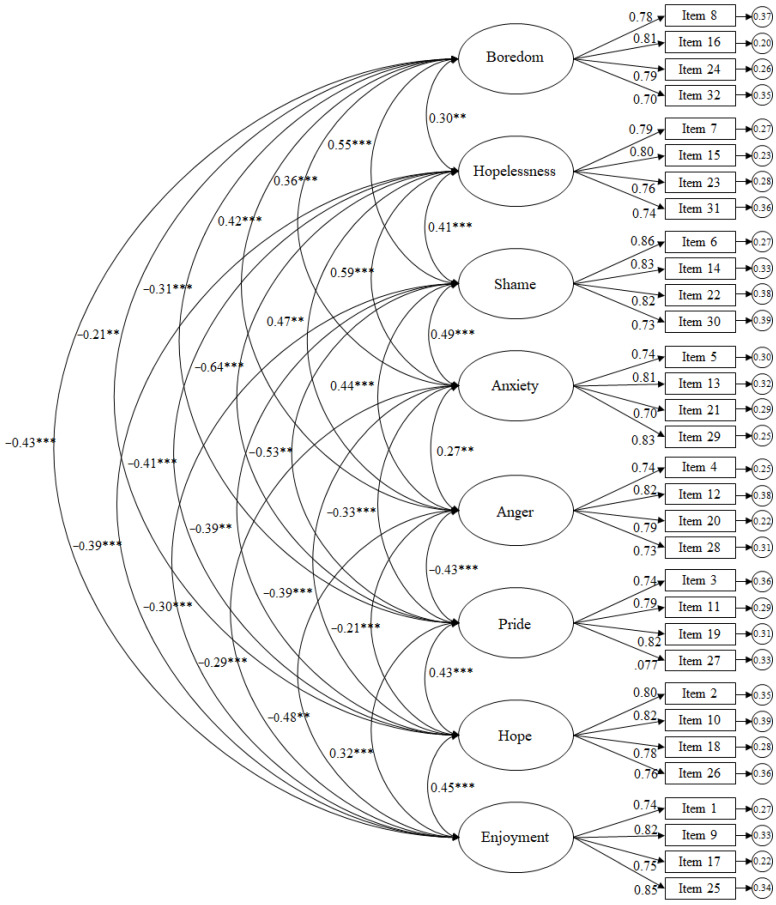
CFA of the AEQ-S focusing on the context towards training. The ellipses represent the factors and the rectangles represent the different items. The residual variances are shown in the small circles. Note: *** *p* < 0.001; ** *p* < 0.01.

The residual variances are shown in the small circles. Results reflected in the CFA for the learning-related emotions scale revealed the following scores (Figure 2): χ^2^ (436, N = 468) = 1078.79, *p* = 0.001; χ^2^/df = 2.47; CFI = 0.96; TLI = 0.96; IFI = 0.96; RMSEA = 0.057 (95% CI = 0.053–0.060); SRMR = 0.041. To evaluate local fit, standardised residuals were examined, revealing that all absolute values were below 2.58, indicating no major item-level misfit. The factor correlation matrix showed moderate correlations between factors, supporting discriminant validity while remaining consistent with the theoretical model. Standardised regression weights were statistically significant (*p* < 0.001), ranging from 0.70 to 0.86.

**Figure 3:**
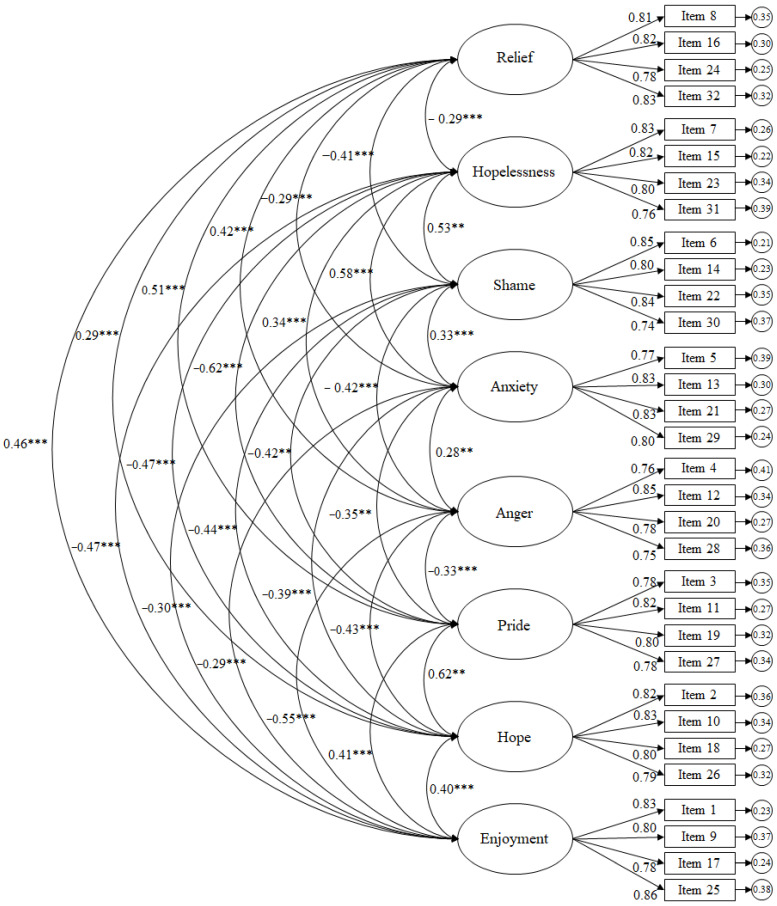
CFA of the AEQ-S focusing on the context towards competition. The ellipses represent the factors and the rectangles represent the different items. The residual variances are shown in the small circles. Note: *** *p* < 0.001; ** *p* < 0.01.

The results reflected in the CFA for the scale of emotions related to competitions revealed the following scores (Figure 3): χ^2^ (436, N = 468) = 1000.29, *p* = 0.001; χ^2^/df = 2.29; CFI = 0.95; TLI = 0.95; IFI = 0.95; RMSEA = 0.055 (95% CI = 0.050–0.061); SRMR = 0.036. To evaluate local fit, standardised residuals were examined, revealing that all absolute values were below 2.58, indicating no major item-level misfit. The factor correlation matrix showed moderate correlations between factors, supporting discriminant validity while remaining consistent with the theoretical model. Standardised regression weights were statistically significant (*p* < 0.001), ranging from 0.74 to 0.86.

### 3.3. Descriptive Analyses and Bivariate Correlations

The descriptive statistics (mean and standard deviation), the reliability analyses (Cronbach’s omega index and alpha), the temporal stability analysis (intraclass correlation index [ICC]), and the bivariate correlations can be seen in Table 4. Furthermore, Table 4 shows the discriminant validity analyses through the HTMT analysis under the diagonal. The results revealed that there is no overlap between the factors of each of the scales as all the scores were below 0.90 (Heterotrait–Monotrait; [36,37]).

On the other hand, in Supplementary Tables S1 and S2 shows the bivariate correlations between emotions related to training, competition and learning. Results have shown that each of the emotions belonging to the three scales have shown discriminant validity, as scores have been below 0.85 and with HTMT scores below 0.90 [36,37].

### 3.4. Linear Regression Analysis

Supplementary Table S3 shows the linear regression analysis in which each of the emotions, including training-related, competition-related, and learning-related (separately), was related to resilience. This analysis tries to reflect the association of the scale, where the results showed that negative emotions (boredom, hopelessness, anxiety, anger and shame) were negatively related to resilience, while positive emotions (pride, calmness, hope, and enjoyment) were positively related to resilience.

## 4. Discussion

The main objective of this study was to adapt the short version of the AEQ [14] designed and validated by Bieleke et al. [9] in an academic context to the sport context. For this purpose, an exploratory factor analysis was first performed, in which the results obtained showed an adequate distribution of the items for each context evaluated (learning, training sessions, and competition), with Kaiser–Meyer–Olkin values and Bartlett’s statistics showing acceptable fit indices. The explored factor structures coincide with the structures proposed by Bieleke et al. [9] and also explored in another research for the context of Secondary Education in the subject of Physical Education [38], where the scales for learning situations and for classes obtained the same distribution of items, in the case of the exploration for the questionnaire that addresses emotions in an exam situation, this was not explored in the study because the performance of written exams in the subject of physical education is unusual [39].

In addition, three CFAs were carried out for each of the scales, which reported adequate and significant fit indices, both for the learning scale and for the training session and competition scales. These results are in line with those obtained in the original version of the AEQ-S by Bileke et al. [9] and also confirmed later in the study by Trigueros & Aguilar-Parra [38] in Physical Education classes with Secondary Education students. Regarding the reliability reported by the scales, adequate internal consistency indices were found, with values > 0.70 for Cronbach’s alpha and McDonald’s Omega, in each of the eight factors for each of the three scales, these values being aligned with those obtained by the AEQ-S in its original version and in previous research [38]. This study also incorporates the evaluation of the temporal stability of the scales, which was not evaluated in the development by Bileke et al. [9] of the AEQ-S, obtaining adequate values for the intraclass correlation indexes, similar to those reported for the learning and class scales in the study developed by Trigueros and Aguilar-Parra [38].

On the contrary, the bivariate correlation analyses revealed values that align with the expected behaviour according to the theoretical relationships that should be evident among the factors comprising each scale. Hence, significant relationships were observed among all of them, with positive correlations corresponding to emotions classified, according to the taxonomy proposed by Pekrun et al. [4,8,14], as having positive and activating valence (fun, hope, pride, and calm), and negative correlations associated with emotions characterised by negative and deactivating valence (anger, hopelessness, shame, anxiety, and boredom), with the combination of both indicating a negative connotation. These results are in line with those obtained in studies of the long and short version of the scale [4,8,9,14,21,24,38].

Finally, the results of the present study have shown a positive relationship between positive emotions, those related to training, competition, and learning experiences, with resilience. The positive relationship found between positive emotions and resilience is theoretically based on the Broaden-and-Build Theory. According to this framework, emotions such as enjoyment and hope not only generate momentary well-being, but also expand the athlete’s repertoire of thoughts and actions, allowing them to build lasting personal resources. In sport, these resources translate into greater resilience, making it easier for athletes to interpret stressful situations (such as injury or defeat) as surmountable challenges rather than insurmountable threats. Conversely, negative emotions tend to narrow the focus of attention and deplete coping resources, which explains their inverse correlation with athletes’ resilience. In this sense, findings align with previous studies demonstrating that positive emotions can foster positive adaptive outcomes in sports [40], while negative emotions exert a negative influence on these adaptive processes [41,42,43]. These results stem from the fact that resilience is a construct referring to a set of personal competencies that constitute the human capacity to overcome unfavourable and stressful situations, as well as to sustain the positive growth of the individual resulting from the achievement of their personal and athletic challenges [44]. Therefore, the experiences generated during sports practice are crucial, as the athlete’s emotional and psychological well-being plays a significant role in their ability to assimilate information and learn from it [45,46]. Thus, if the training sessions are enjoyable, fun, and the coach dedicates time to each athlete, greater athlete engagement will be achieved, leading to an increase in resilience [47]. However, if the training sessions are tedious, monotonous, and boring, athletes are likely to develop an aversion to training, resulting in lower engagement [48].

A relevant aspect for the generalisation of these findings is the diversity of the sample, which included both team sports (football, basketball, volleyball, handball) and individual sports (athletics). Although emotions of achievement such as enjoyment or anxiety are universal in the competitive context, their manifestation may vary depending on the nature of the sport. In team sports, emotions are often mediated by interpersonal factors and collective efficacy, while in individual sports the focus of control and value tends to be more self-referential. The results of this study suggest that the adapted AEQ-S has the necessary sensitivity to capture these experiences in both contexts, although future research should perform multigroup invariance analyses to confirm whether the factor structure remains strictly identical between individual and team athletes.

### 4.1. Limitation and Prospective

With regard to the limitations of the study, it should be taken into account that the results obtained here correspond to a first study in which the psychometric properties of the AEQ-S are analysed in a sports context, and these data should be taken as preliminary for this context. It should be emphasised that the study relied on a convenience sample of young athletes, which may limit the generalizability of the findings to other populations, such as older athletes, elite competitors, or participants in less-studied sports disciplines. This sample selection bias could influence the observed correlations and the stability of the factorial structure of the AEQ-S. Furthermore, the cross-sectional design precludes causal inference between achievement emotions and related variables, limiting the ability to examine temporal dynamics or developmental changes. Additionally, the exclusive use of self-report measures collected at a single time point raises the possibility of common method bias, which may artificially inflate relationships among factors and affect the model fit. Future research should address these limitations by including more diverse and representative samples, adopting longitudinal designs, and combining multiple assessment methods to enhance the robustness and external validity of the findings. Finally, it should be noted that it would have been valuable to provide more information about the participants (for example, years of experience; frequency of exercise, whether they are international, etc.).

### 4.2. Conclusions

This study demonstrates that the AEQ-S is a psychometrically robust instrument for assessing achievement emotions in young athletes, showing satisfactory factorial validity, internal consistency, and discriminant reliability. Beyond confirming its reliability, the findings contribute theoretically by highlighting the specific structure and dynamics of achievement emotions in sport contexts, differentiating them from academic settings and reinforcing the relevance of Pekrun’s control-value framework within athletic populations. Practically, the AEQ-S can be utilised by coaches, sport psychologists, and researchers to systematically monitor athletes’ emotional experiences during training and competition, identify emotional patterns that may affect motivation or performance, and design targeted psychological interventions aimed at enhancing well-being, persistence, and performance outcomes. For example, positive emotion profiles could inform reinforcement strategies, whereas elevated levels of anxiety or hopelessness could prompt individualised support or adjustments in training load. By providing a validated, context-specific tool, this study facilitates both empirical research and applied practice, bridging theoretical insights on achievement emotions with actionable strategies in real-world sports settings.

## Figures and Tables

**Table 1:** Exploratory factorial analysis (emotions learning).

Item	Enjoyment	Hope	Pride	Anger	Anxiety	Shame	Hopelessness	Boredom
1	0.74							
9	0.75							
17	0.72							
25	0.77							
2		0.70						
10		0.76						
18		0.74						
26		0.75						
3			0.76					
11			0.72					
19			0.77					
27			0.74					
4				0.75				
12				0.73				
20				0.79				
28				0.71				
5					0.76			
13					0.72			
21					0.72			
29					0.75			
6						0.77		
14						0.73		
22						0.75		
30						0.77		
7							0.79	
15							0.74	
23							0.80	
31							0.74	
8								0.74
16								0.72
24								0.75
32								0.77

Note: Factor loadings of emotion learning subscale.

**Table 2:** Exploratory factor analysis (training).

Item	Enjoyment	Hope	Pride	Anger	Anxiety	Shame	Hopelessness	Boredom
1	0.77							
9	0.69							
17	0.73							
25	0.75							
2		0.74						
10		0.72						
18		0.79						
26		0.71						
3			0.73					
11			0.70					
19			0.76					
27			0.73					
4				0.73				
12				0.75				
20				0.70				
28				0.72				
5					0.76			
13					0.74			
21					0.72			
29					0.75			
6						0.76		
14						0.74		
22						0.73		
30						0.71		
7							0.72	
15							0.69	
23							0.74	
31							0.71	
8								0.69
16								0.67
24								0.74
32								0.71

Note: Factor loadings of training emotion subscale.

**Table 3:** Exploratory factor analysis (competition).

Item	Enjoyment	Hope	Pride	Anger	Anxiety	Shame	Hopelessness	Relief
1	0.77							
9	0.72							
17	0.67							
25	0.76							
2		0.72						
10		0.74						
18		0.77						
26		0.79						
3			0.73					
11			0.71					
19			0.77					
27			0.74					
4				0.70				
12				0.69				
20				0.73				
28				0.75				
5					0.71			
13					0.75			
21					0.77			
29					0.73			
6						0.73		
14						0.72		
22						0.69		
30						0.68		
7							0.74	
15							0.70	
23							0.69	
31							0.77	
8								0.69
16								0.72
24								0.66
32								0.73

Note: Factor loadings of competition emotion subscale.

**Table 4:** Descriptive statistics, reliability analysis, HTMT, bivariate correlations, and temporal stability analysis.

Training-Related Emotions													
Factors	M	SD	α	ω	ICC	1	2	3	4	5	6	7	8
1. Boredom	2.10	0.81	0.80	0.81	0.90	-	0.41 ***	0.38 ***	0.52 **	0.28 ***	−0.17 **	−0.22 ***	−0.67 ***
2. Hopelessness	1.79	0.73	0.84	0.84	0.89	0.46	-	0.27 **	0.33 ***	0.48 **	−0.24 ***	−0.58 ***	−0.49 ***
3. Shame	1.63	0.71	0.79	0.79	0.92	0.44	0.34	-	0.50 ***	0.23 **	−0.21 **	−0.43 ***	−0.36 **
4. Anxiety	1.73	1.03	0.83	0.83	0.89	0.60	0.39	0.58	-	0.55 ***	−0.19 ***	−0.27 *	−0.24 **
5. Anger	1.86	0.98	0.88	0.88	0.91	0.34	0.52	0.30	0.61	-	−0.34 **	−0.46 **	−0.55 **
6. Pride	3.25	1.09	0.80	0.81	0.88	−0.22	−0.30	−0.27	−0.25	−0.41	-	0.34 ***	0.12 ***
7. Hope	3.77	0.86	0.82	0.82	0.87	−0.30	−0.64	−0.50	−0.32	−0.51	0.40	-	0.38 ***
8. Enjoyment	4.08	0.69	0.86	0.87	0.92	−0.73	−0.55	−0.44	−0.31	−0.61	0.18	0.46	-
Emotions-Related to Learning													
Factors	M	SD	α	ω	CCI	1	2	3	4	5	6	7	8
1. Boredom	2.20	0.76	0.83	0.84	0.84	-	0.37 **	0.25 ***	0.36 ***	0.48 **	−0.26 ***	−0.32 **	−0.59 ***
2. Hopelessness	1.68	0.82	0.79	0.80	0.87	0.46	-	0.34 **	0.29 **	0.41 **	−0.48 **	−0.38 **	−0.51 **
3. Shame	2.33	0.91	0.85	0.85	0.85	0.28	0.40	-	0.34 ***	0.38 ***	−0.32 *	−0.35 **	−0.42 ***
4. Anxiety	2.09	1.01	0.83	0.83	0.88	0.44	0.36	0.39	-	0.31 ***	−0.37 **	−0.52 ***	−0.38 ***
5. Anger	1.57	0.85	0.84	0.84	0.91	0.56	0.49	0.45	0.37	-	−0.54 ***	−0.41 ***	−0.26 ***
6. Pride	3.81	0.79	0.82	0.82	0.82	−0.31	−0.53	−0.38	−0.43	−0.60	-	0.29 ***	0.33 ***
7. Hope	3.74	0.83	0.78	0.79	0.88	−0.39	−0.45	−0.41	−0.56	−0.47	0.35	-	0.37 ***
8. Enjoyment	3.88	0.82	0.87	0.87	0.85	−0.64	−0.57	−0.50	−0.46	−0.31	0.40	0.48	-
Competition-Related Emotions													
Factors	M	SD	α	ω	CCI	1	2	3	4	5	6	7	8
1. Anger	2.04	0.85	0.81	0.81	0.87	-	0.24 ***	0.11 *	0.47 ***	−0.59 ***	−0.33 **	−0.26 ***	−0.22 **
2. Hopelessness	1.55	1.12	0.77	0.78	0.84	0.30	-	0.39 **	0.27 **	−0.14 **	−0.31 **	−0.62 ***	−0.37 **
3. Shame	1.99	1.04	.84	0.85	0.86	0.18	0.44	-	0.55 ***	0.19	−0.17 *	−0.35 **	−0.22 ***
4. Anxiety	2.13	1.29	0.83	0.84	0.88	0.54	0.32	0.62	-	−0.63 ***	−0.22 **	−0.44 ***	−0.18 ***
5. Relief	3.04	0.88	0.80	0.81	0.85	−0.64	−0.20	0.24	−0.70	-	0.32 **	0.25 *	0.38 ***
6. Pride	3.32	1.07	0.79	0.79	0.86	−0.40	−0.37	−0.24	−0.28	0.38	-	0.39 ***	0.28 **
7. Hope	3.52	0.81	0.82	0.82	0.83	−0.32	−0.70	−0.42	−0.51	0.31	0.45	-	0.41 ***
8. Enjoyment	3.05	0.93	0.78	0.79	0.87	−0.27	−0.45	−0.28	−0.25	0.43	0.35	0.48	-

Note: *** *p*< 0.001; ** *p*< 0.01; * *p*< 0.05.

## Data Availability

The data availability is not available unless a justified request is made to the contact author because the data matrix cannot be disseminated without participants’ permission.

## Supplementary Materials

The following supporting information can be downloaded at https://www.mdpi.com/article/10.3390/brainsci16010046/s1, Table S1: Bivariate correlations between each scale; Table S2: HTMT scores between each scale; Table S3: Linear regression analysis.

## Appendix A. Sports Learning Emotions

The following scales have been validated in Spanish:

**Enjoyment**

1. I like the challenge of learning new skills.

9. I enjoy learning when I make progress in my skills and abilities.

17. I am so happy with the progress I have made that it motivates me to keep working.

25. When I make progress in my skills, it gives me a high.

**Hope**

2. I feel confident when I am successful in training.

10. I am confident that I will be able to master the challenges I face.

18. I feel optimistic that I will make progress in developing my skills.

26. I am motivated by my sense of confidence.

**Pride**

3. I feel proud of myself.

11. I believe I can be proud of my achievements.

19. I am highly motivated because I am proud of my achievements.

27. When I excel in training or during competition, I am proud of myself.

**Anger**

4. Training sessions irritate me.

12. It annoys me to have to train.

20. I get so angry that I want to throw it all away and quit.

28. When I stand still for long periods of time, my irritation makes me restless.

**Anxiety**

5. I become tense and nervous while doing the exercises.

13. I worry about whether I will be able to cope with the challenges of training or competing.

21. While training I feel like distracting myself to reduce my anxiety.

29. Worrying about not completing the exercises makes me sweat.

**Shame**

6. I feel embarrassed.

14. I feel ashamed when I realise that I lack ability.

22. Because I have had a lot of problems in doing the exercises, I avoid talking about it.

30. When someone notices how unskillful I am, I avoid eye contact.

**Hopelessness**

7. I feel helpless.

15. I resign myself to the fact that I do not have the ability to succeed.

23. I feel so incapable that I cannot devote all my efforts to improving my skills.

31. My lack of confidence causes me to burn out before I even start.

**Boredom**

8. Training sessions bore me.

16. Training sessions are so boring that I find myself daydreaming.

24. I would rather stop learning new skills because it is so boring.

32. As I learn new skills, I seem to drift away because it is so boring.

**Spanish Version**

**Diversión**

1. Me gusta el reto de aprender nuevas habilidades.

9. Disfruto cuando aprendo progreso en mis habilidades y destrezas.

17. Estoy tan contento con los progresos que he hecho que me motiva a seguir trabajando.

25. Cuando progreso en mis habilidades, me da un subidón.

**Esperanza**

2. Me siento confiado cuando tengo éxito en los entrenamientos.

10. Tengo confianza en que podré dominar los retos a los que me enfrente.

18. Me siento optimista de que voy a progresar en el desarrollo de mis habilidades.

26. Mi sensación de confianza me motiva.

**Orgullo**

3. Me siento orgulloso de mí mismo.

11. Creo que puedo estar orgulloso de mis logros.

19. Estoy muy motivado porque estoy orgulloso de mis logros.

27. Cuando destaco en los entrenamientos o durante la competición, siento orgullo de mí mismo.

**Ira**

4. Los entrenamientos me irritan.

12. Me molesta tener que entrenar.

20. Me enfado tanto que me dan ganas de tirarlo todo y dejarlo.

28. Cuando estoy parado durante mucho tiempo, mi irritación me hace estar inquieto.

**Ansiedad**

5. Me pongo tenso y nervioso mientras hago los ejercicios.

13. Me preocupa si voy a ser capaz de hacer frente a los retos que supone entrenar o competir.

21. Mientras entreno me apetece distraerme para reducir mi ansiedad.

29. La preocupación por no completar los ejercicios me hace sudar.

**Vergüenza**

6. Me siento avergonzado.

14. Siento vergüenza cuando me doy cuenta de que me falta capacidad.

22. Como he tenido muchos problemas para realizar los ejercicios, evito hablar de ello.

30. Cuando alguien se da cuenta de lo poco habilidoso que soy, evito el contacto visual.

**Desesperanza**

7. Me siento impotente.

15. Me resigno al hecho de que no tengo la capacidad de alcanzar el éxito.

23. Me siento tan incapaz que no puedo dedicar todo mi esfuerzo a mejorar mis habilidades.

31. Mi falta de confianza hace que me agote incluso antes de empezar.

**Aburrimiento**

8. Los entrenamientos me aburren.

16. Los entrenamientos son tan aburrido que me encuentro soñando despierto.

24. Prefiero dejar de aprender habilidades nuevas por lo aburrido que es.

32. Mientras aprendo nuevas habilidades, parece que me alejo porque es muy aburrido.

## Appendix B. Training Sessions Emotion

**Enjoyment**

1. I like to train.

9. I look forward to learning a lot in training.

17. I am motivated to go to training because it is exciting.

25. I like training so much that it fills me with energy.

**Hope**

2. I have confidence in myself when I go training.

10. I am full of hope.

18. I am confident because I understand the trainings.

26. I am confident that I will understand the trainings, and that, motivates me.

**Pride**

3. I am proud of myself.

11. I believe that I can be proud of what I can do.

19. Because I take pride in my accomplishments, I am motivated to continue.

27. When I do well in training, my heart beats with pride.

**Anger**

4. I am angry.

12. When I think of the time I waste in training, I get angry.

20. I wish I didn’t have to attend training sessions because I get angry.

28. I feel anger welling up in me.

**Anxiety**

5. I feel nervous at training sessions.

13. Even before the class, I worry about whether I will be able to understand the tactical elements.

21. Because I am very nervous, I prefer to skip training sessions.

29. I get tense in training.

**Shame**

6. I feel embarrassed.

14. When I say something in training I feel I am being ridiculous.

22. After I say something in training, I would like to crawl into a hole and hide.

30. Because I am embarrassed, I become tense and inhibited.

**Hopelessness**

7. I feel hopeless.

15. I have lost all hope of understanding the tactical elements.

23. Because I have given up, I have no energy to go to the training.

31. I feel so hopeless that all my energy is drained.

**Boredom**

8. I get bored.

16. The workouts bore me.

24. I think about what else I could be doing instead of being in this boring workout.

32. I get restless because I can’t wait for the workout to end.

**Spanish Version**

**Diversión**

1. Me gusta entrenar.

9. Tengo ganas de aprender mucho en los entrenamientos.

17. Me motiva ir a entrenar porque es emocionante.

25. Me gusta tanto entrenar que me llena de energía.

**Esperanza**

2. Tengo confianza en mí mismo cuando voy a entrenar.

10. Estoy lleno de esperanza.

18. Tengo confianza porque entiendo los entrenamientos.

26. Estoy seguro de que voy a entender los entrenamientos, y eso, me motiva.

**Orgullo**

3. Estoy orgulloso de mí mismo.

11. Creo que puedo estar orgulloso de lo que puedo hacer.

19. Como me enorgullezco de mis logros, me siento motivado para continuar.

27. Cuando me va bien en los entrenamientos, mi corazón palpita de orgullo.

**Enfado**

4. Estoy enfadado.

12. Cuando pienso en el tiempo que pierdo en los entrenamientos me enfado.

20. Me gustaría no tener que asistir a los entrenamientos porque me enfada.

28. Siento que la ira brota en mí.

**Ansiedad**

5. Me siento nervioso en los entrenamientos.

13. Incluso antes de la clase, me preocupa si seré capaz de entender los elementos tácticos.

21. Como estoy muy nervioso, prefiero saltarme los entrenamientos.

29. Me pongo tenso en los entrenamientos.

**Vergüenza**

6. Me siento avergonzado.

14. Cuando digo algo en los entrenamientos siento que estoy haciendo el ridículo.

22. Después de decir algo en los entrenamientos, me gustaría meterme en un agujero y esconderme.

30. Como me da vergüenza, me pongo tenso y me inhibo.

**Desesperanza**

7. Me siento desesperado.

15. He perdido toda esperanza de entender los elementos tácticos.

23. Como me he rendido, no tengo energía para ir a los entrenamientos.

31. Me siento tan desesperado que toda mi energía está agotada.

**Aburrimiento**

8. Me aburro.

16. Los entrenamientos me aburre.

24. Pienso en qué otra cosa podría estar haciendo en lugar de estar en este aburrido entrenamiento.

32. Me pongo inquieto porque no puedo esperar a que termine el entrenamiento.

## Appendix C. Competition Emotion

**Enjoyment**

1. I enjoy competing.

9. For me, competition is a challenge that I enjoy.

17. Because I enjoy preparing for competition, I feel motivated to do more than I need to do.

25. Before I compete, I feel a sense of impatience.

**Hope**

2. I am optimistic and believe that everything will go well.

10. I have a lot of confidence in myself.

18. I think about my competition with optimism.

26. My confidence motivates me to prepare well.

**Pride**

3. I am proud of myself.

11. I am proud of my performance during the competition.

19. Pride in my knowledge fuels my efforts during the competition.

27. After my success during the competition, I feel very proud.

**Anger**

4. I get angry.

12. I get angry because of the coach’s tactics.

20. I would like to scold the coach.

28. Anger makes my blood rush to my head.

**Anxiety**

5. I am very nervous.

13. I worry that the competition will be too difficult.

21. I get so nervous that I wish I didn’t have to compete.

29. At the beginning of the competition, my heart starts pounding.

**Shame**

6. I feel ashamed.

14. I am ashamed because I cannot respond during the competition.

22. I am so ashamed that I want to run and hide.

30. Because, I am ashamed my pulse quickens.

**Hopelessness**

7. I feel hopeless.

15. I begin to think that no matter how hard I try, I will fail.

23. I feel like giving up.

31. I feel so resigned that I have no energy.

**Relief**

8. After the competition, I feel relieved.

16. After the competition, I feel liberated.

24. After the competition, the tension in my stomach dissipates.

32. After the competition, I can finally breathe easy again.

**Spanish Version**

**Disfrute**

1. Disfruto compitiendo.

9. Para mí la competición es un reto que me divierte.

17. Como disfruto preparándome para la competición, me siento motivado para hacer más de lo necesario.

25. Antes de competir, tengo una sensación de impaciencia.

**Esperanza**

2. Soy optimista y creo que todo saldrá bien.

10. Tengo mucha confianza en mí mismo.

18. Pienso en mi competición con optimismo.

26. Mi confianza me motiva para prepararme bien.

**Orgullo**

3. Estoy orgulloso de mí mismo.

11. Estoy orgulloso de mi desempeño durante la competición.

19. El orgullo por mis conocimientos alimenta mis esfuerzos durante la competición.

27. Después de mi éxito durante la competición me siento muy orgulloso.

**Enfado**

4. Me enfado.

12. Me enfado por las tácticas del entrenador.

20. Me gustaría regañar al entrenador.

28. La ira hace que la sangre se me suba a la cabeza.

**Ansiedad**

5. Estoy muy nervioso.

13. Me preocupa que sea muy difícil la competición.

21. Me pongo tan nervioso que desearía no tener que competir.

29. Al principio de la competición, mi corazón empieza a latir con fuerza.

**Vergüenza**

6. Siento vergüenza.

14. Me avergüenzo porque no puedo responder durante la competición.

22. Me da tanta vergüenza que quiero correr y esconderme.

30. Porque me avergüenzo se me acelera el pulso.

**Desesperanza**

7. Me siento desesperanzado.

15. Empiezo a pensar que por mucho que me esfuerce fracasaré.

23. Me dan ganas de rendirme.

31. Me siento tan resignado que no tengo energía

**Relajación**

8. Después de la competición siento alivio.

16. Después de la competición me siento liberado.

24. Después de la competición la tensión en mi estómago se disipa.

32. Después de la competición por fin puedo volver a respirar tranquilo.
