# Supplementary material for: Understanding Athlete Emotions: A Psychometric Approach to the AEQ-S in Sports
Source: Brain Sci. 2025 Dec 29;16(1):46. doi: 10.3390/brainsci16010046 (PMC12838535; doi:10.3390/brainsci16010046)
Supplement: Supplementary file 1 [file brainsci-16-00046-s001.zip › brainsci-4041435-supplementary.pdf]

Supplementary Material

Supplementary Material 1 Bivariate correlations between each scale.

Factors	1. Learning Boredom	2. Learning Hopelessness	3. Learning Shame	4. Learning Anxiety	5. Learning Anger	6. Learning Pride	7. Learning Hope	8. Learning Enjoyment
1. Training Boredom	.67***	.23**	.23*	.42**	.32***	-.41***	-.48***	-.43***
2. Training Hopelessness	.46**	.71***	.30**	.25**	.15*	-.20**	-.36***	-.47***
3. Training Shame	.21***	.33**	.62***	.57***	.36**	-.25**	-.35**	-.32***
4. Training Anxiety	.53***	.48**	.20**	.72***	.34**	-.19*	-.41***	-.32**
5. Training Anger	.60***	.26***	.39**	.36**	.71***	-.30**	-.37**	-.44***
6. Training Pride	-.20**	-.32**	-.52***	-.42***	-.39***	.68***	.34**	.33***
7. Training Hope	-.31***	-.48**	-.03	-.46***	-.26***	.16*	.62***	.52**
8. Training Enjoyment	-.29**	-.30**	-.46***	-.39**	-.11*	.32***	.29***	.74***
Factors	1. Competition Relief	2. Competition Hopelessness	3. Competition Shame	4. Competition Anxiety	5. Competition Anger	6. Competition Pride	7. Competition Hope	8. Competition Enjoyment
1. Training Boredom	-.36**	.31**	.22***	.36**	.45***	-.38**	-.14**	-.60***
2. Training Hopelessness	-.17*	.65***	.30**	.42***	.37**	-.26*	-.55**	-.29**
3. Training Shame	-.30**	.46***	.71***	.45**	.30***	-.19*	-.36***	-.10*
4. Training Anxiety	-.24**	.22**	.36**	.66***	.29**	-.24**	-.23**	-.33**
5. Training Anger	-.57***	.19*	.48***	.21	.68***	-.38***	-.41**	-.40***
6. Training Pride	.25**	-.35***	-.43**	-.12*	-.41***	.75***	.46***	.38***
7. Training Hope	.43***	-.61***	-.55***	-.30**	-.09	.52***	.73***	.29*
8. Training Enjoyment	.29***	-.26**	-.22*	-.43***	-.36**	.37**	.28*	.76***
Factors	1. Learning Boredom	2. Learning Hopelessness	3. Learning Shame	4. Learning Anxiety	5. Learning Anger	6. Learning Pride	7. Learning Hope	8. Learning Enjoyment
1. Competition Relief	-.43***	-.25*	-.24**	-.51***	-.43**	.38***	.25**	.18*
2. Competition Hopelessness	.21**	.72***	.42**	.26**	.42***	-.21***	-.59***	-.31**
3. Competition Shame	.26***	.30**	.78***	.16*	.25*	-.50***	-.25**	-.45***
4. Competition Anxiety	.34**	.25**	.32**	.69***	.53***	-.12*	-.34**	-.22**
5. Competition Anger	.40**	.17*	.36***	.35***	.62***	-.08	-.57***	-.15**
6. Competition Pride	-.37***	-.29**	-.25**	-.27**	-.20*	.74***	.19*	.26**
7. Competition Hope	-.25**	-.61***	-.31***	-.39***	-.25**	.47**	.62***	.24**
8. Competition Enjoyment	-.66***	-.21**	-.14*	-..42***	-.36***	.35**	.26*	.77***

Note: ***p< .001; **p< .01; *p<.05.

Supplementary Material 2 HTMT scores between each scale.

Factors	1. Learning Boredom	2. Learning Hopelessness	3. Learning Shame	4. Learning Anxiety	5. Learning Anger	6. Learning Pride	7. Learning Hope	8. Learning Enjoyment
1. Training Boredom	.72	.26	.29	.50	.40	-.49	-.55	-.49
2. Training Hopelessness	.52	.76	.35	.32	.22	-.28	-.43	-.52
3. Training Shame	.28	.39	.66	.65	.41	-.32	-.41	-.39
4. Training Anxiety	.57	.55	.27	.78	.40	-.27	-.47	-.37
5. Training Anger	.66	.35	.45	.41	.79	-.39	-.41	-.52
6. Training Pride	-.26	-.37	-.58	-.48	-.46	.75	.40	.41
7. Training Hope	-.35	-.53	-.10	-.52	-.32	.23	.70	.60
8. Training Enjoyment	-.34	-.36	-.58	-.45	-.19	.40	.36	.78
**Factors**	**1. Competition Relief**	**2. Competition Hopelessness**	**3. Competition Shame**	**4. Competition Anxiety**	**5. Competition Anger**	**6. Competition Pride**	**7. Competition Hope**	**8. Competition Enjoyment**
1. Training Boredom	-.41	.37	.28	.43	.50	-.43	-.20	-.65
2. Training Hopelessness	-.23	.71	.35	.46	.42	-.32	-.60	-.34
3. Training Shame	-.36	.48	.77	.49	.36	-.26	-.42	-.15
4. Training Anxiety	-.30	.29	.42	.72	.34	-.30	-.30	-.36
5. Training Anger	-.62	.25	.52	.26	.73	-.42	-.46	-.45
6. Training Pride	.31	-.42	-.50	-.17	-.46	.79	.51	.42
7. Training Hope	.48	-.67	-.61	-.36	-.15	.56	.77	.33
8. Training Enjoyment	.34	-.34	-.28	-.50	-.42	.42	.33	.80
**Factors**	**1. Learning Boredom**	**2. Learning Hopelessness**	**3. Learning Shame**	**4. Learning Anxiety**	**5. Learning Anger**	**6. Learning Pride**	**7. Learning Hope**	**8. Learning Enjoyment**
1. Competition Relief	-.48	-.30	-.29	-.56	-.47	.43	.30	.26
2. Competition Hopelessness	.26	.76	.47	.31	.48	-.29	-.63	-.39
3. Competition Shame	.31	.34	.83	.20	.30	-.55	-.30	-.51
4. Competition Anxiety	.38	.30	.38	.73	.57	-.18	-.39	-.28
5. Competition Anger	.46	.23	.41	.40	.66	-.15	-.52	-.20
6. Competition Pride	-.41	-.33	-.30	-.33	-.24	.78	.24	.31
7. Competition Hope	-.30	-.65	-.36	-.42	-.30	.52	.68	.28
8. Competition Enjoyment	-.71	-.26	-.19	-..45	-.41	.41	.32	.81

Supplementary Material 3 Linear Regression Analysis.

	F	R^2	*β (CI)*	*t*
Training Related Emotions				
	28.30	.49***		
1. Boredom			-.22 (-.34--.18)	-2.45**
2. Hopelessness			-.35 (-.44--.27)	-1.99***
3. Shame			-.28 (-.37--.19)	-1.84**
4. Anxiety			-.26 (-.39 -.22)	-1.02**
5. Anger			-.30 (-.41 -.27)	-.53**
6. Pride			.32 (.28 .47)	1.45**
7. Hope			.37 (.29 - .55)	1.12**
8. Enjoyment			.53 (.46 - .63)	2.88***
Emotions Related to Learning				
	31.06	.34***		
1. Boredom			-.38 (-.46--.25)	-2.11***
2. Hopelessness			-.47 (-.57--.26)	-2.87***
3. Shame			-.40 (-.48--.35)	-1.83***
4. Anxiety			-.41 (-.44--.32)	-1.70**
5. Anger			-.46 (-.52--.38)	-.76**
6. Pride			.40 (.36-.47)	1.35***
7. Hope			.43 (.38-.48)	2.21***
8. Enjoyment			.44 (.36-.47)	1.88**
Competition-Related Emotions				
	49.53	.51***		
1. Anger			-.35 (-.43--.24)	-1.88**
2. Hopelessness			-.50 (-.56--.47)	-2.46***
3. Shame			-.39 (-.48--.34)	-1.32***
4. Anxiety			-.32 (-.43--.24)	-1.17**
5. Relief			.28 (.17-.34)	1.08***
6. Pride			.40 (.33-.49)	1.45***
7. Hope			.37 (.28-.46)	2.11**
8. Enjoyment			.52 (.44-.56)	1.66**

***$p < .001$; **$p < .01$ Note: CI = confidence interval 95%.
